# Sequential treatment of teriparatide and alendronate versus alendronate alone for elevation of bone mineral density and prevention of refracture after percutaneous vertebroplasty in osteoporosis: a prospective study

**DOI:** 10.1007/s40520-023-02342-w

**Published:** 2023-01-28

**Authors:** Dazhi Yang, Jie Tan, Yufeng Long, Kang Huang, Weidong Han, Min Wang, Shizhuang Zhu, Shutong Zeng, Weihong Yi

**Affiliations:** 1 Department of Spinal Surgery, The 6th Affiliated Hospital of Shenzhen University Health Science Center, Shenzhen, 518052 Guangdong China; 2 Department of Orthopedics & Orthopedic Institute, West China Hospital, Sichuan University, Chengdu, 610041 Sichuan China; 3 Department of Orthopedics, Traumatology, Prince of Wales Hospital, The Chinese University of Hong Kong, Hong Kong, 999077 China; 4 Department of Pain, The 8th Affiliated Hospital of Sun Yat-Sen University, Shenzhen, 518052 Guangdong China; 5 Department of Orthopedics, Shenzhen Nanshan Hospital Affiliated to Guangdong Medical University, Shenzhen, 518052 Guangdong China; 6 Health Science Center, Shenzhen University, Shenzhen, 518000 Guangdong China

**Keywords:** Osteoporosis, Teriparatide, Alendronate, Bone mineral density, Refracture

## Abstract

**Background:**

Percutaneous vertebroplasty was the most common strategy for osteoporotic vertebral compression fracture. However, refracture after vertebroplasty also occurred and bone mineral density (BMD) was one of the main factors associated with refracture after percutaneous vertebroplasty.

**Aims:**

To investigate the efficacy of a short-sequential treatment of teriparatide followed by alendronate on prevention of refracture after percutaneous vertebroplasty in osteoporotic patients, and compare it with the therapy of alendronate alone.

**Methods:**

From January 2018 to January 2020, we recruited 165 female osteoporosis patients after percutaneous vertebroplasty who were assigned into sequential treatment of teriparatide followed by alendronate group (TPTD + ALN group) and alendronate alone group (ALN group). The vertebral fracture occurred during this process was also recorded in both the groups. A total of 105 participants completed the 1-year follow-up. Furthermore, BMD and serum procollagen type I N-terminal propeptide (PINP) and C-terminal cross-linking telopeptide of type I collagen (CTX) were compared between the two groups during 1-year follow-up.

**Results:**

The 105 patients were finally included, with 59 in ALN group and 46 in TPTD + ALN group. During 1-year follow-up, the vertebral refracture rate in TPTD + ALN group was much lower than that in ALN group (2.2% vs. 13.6%, *p* < 0.05). At 12 months, the BMDs at lumbar in TPTD + ALN group were significantly elevated when compared to the ALN group (0.65 ± 0.10 vs. 0.57 ± 0.07, *p* < 0.001).

**Discussion and conclusion:**

A short-sequential administration of teriparatide followed by alendronate was more effective in elevating the BMD and decreasing the refracture rate at 12-month follow-up, compared to the counterpart with alendronate alone.

## Introduction

Osteoporotic vertebral compression fracture (OVCF) caused by osteoporosis remained as one of the dominating health problems for the elderly. It was reported that approximately 20% of senior populations over 70 years and 16% of postmenopausal women suffered from such disease [1]. Percutaneous vertebroplasty [2], spine jack system [3], and KIVA system [4] were all the adopted methods to treat OVCF. Among them, percutaneous vertebroplasty was the most commonly used strategy, due to fast pain relief, efficient vertebral height restoration, and acceptable Cobb’s angle maintenance. Nevertheless, refracture after this operation would possibly develop in some cases, and studies demonstrated that low mineral density in those patients were the most accountable culprit, especially in postmenopausal women [1, 5]. Therefore, strategies to treat osteoporosis would be beneficial in reducing the risk of developing refracture in those patients. Osteoporosis treatment strategies involved assessing risk factors for fracture, nutrition and lifestyle, anti-osteoporosis drugs, osteoporosis management, etc. [6].

Traditionally, estrogen [7], bisphosphonate [8], calcitonin [9], or some monoclonal antibodies [10] were all anti-resorptive agents which could compellingly reduce the rate of bone resorption [11], while recombinant human parathyroid hormones (such as teriparatide) were anabolic drugs that could significantly improve BMD (bone mineral density) and bone strength [12, 13]. Compared with anti-reabsorptive and anabolic drugs alone, the effect of anabolic drugs is much more obvious [6]. Teriparatide, one of anabolic drugs, was approved to use since no more than 2 years [12]. Even though teriparatide was reported to be effective in promoting the anabolic process of bone turnover which would be helpful for increasing the BMD for osteoporotic patients, the drawbacks such as high expenditure, daily administration by subcutaneous injection, and bone mass loss after discontinuation greatly limited its clinic applications [13, 14]. A cost-effective and effective treatment strategy for osteoporosis is a challenge [15]. Meanwhile, effective transdisciplinary management of osteoporosis patients can reduce postoperative complications [16]. The fracture liaison service (FLS), proposed by the International Osteoporosis Foundation, is designed to reduce the rate of refracture of osteoporotic fractures. Therefore, refracture after osteoporotic vertebral compression fracture is a challenge for surgeons.

Recently, some researches showed that sequential administration of parathyroid hormone followed by bisphosphonate was effective in increasing the BMD of osteoporotic patients, in which the followed therapy of bisphosphonate could effectively maintain and elevate the BMD after teriparatide discontinuation [17]. Unfortunately, definite conclusion regarding those findings was still lacking and needed to further clarify.

Bone turnover markers have the potential to provide feedback to patients and prescribers whether the anti-osteoporosis therapies are effective. The International Osteoporosis Foundation has proposed that the reference marker for bone formation should be Serum N-propeptide of type I collagen (PINP) and β-C-terminal telopeptide of type I collagen (CTX) for bone resorption. According to the study, teriparatide can reach the peak expression of PINP osteogenic marker in 6th month [13]. In the present study we investigate the safety and efficacy of applying sequential administration of teriparatide for the initial 6 months followed by alendronate for another 6 months (TPTD + ALN group) for osteoporosis patients, and compared it with the therapy of alendronate alone (ALN group). This study aims to offer some useful decision-making references for clinical physicians to treat osteoporosis, as well as to reduce the occurrence of adverse events such as refracture.

## Methods

### Study design and patients

This prospective study was conducted from January 2018 to January 2020 in two clinic centers in Guangdong province of China (The 6th Affiliated Hospital of Shenzhen University Health Science Center, The 8th Affiliated Hospital of Sun Yat-Sen University). Osteoporosis female patients who had completed percutaneous vertebroplasty were included. All patients had a single vertebral fracture from the thoracic or lumbar vertebrae. A total of 215 primary participants were registered in our two institutes. The inclusion criteria were elderly female patients, aged from 55 to 80 years, fresh vertebral fractures diagnosed by MRI (Magnetic Resonance Imaging) and classified as Genant type I–II, who had completed percutaneous vertebroplasty within 30 days and whose T scores were less than −2.5 confirmed by dual-energy X-ray absorptiometry. The exclusion criteria were patients who had secondary osteoporosis, malignant disease, a history of bisphosphonate and parathyroid hormone prescription, a history of kidney disease and gastrointestinal disorders, such as inflammatory bowel disease and Crohn’s disease, or with medications that could intervene in bone metabolism.

Patients were assigned to TPTD + ALN group or ALN group based on their own choices. In the TPTD + ALN group, participants were administered with daily injection of 20 μg teriparatide for the first 6 months followed by oral administration of 70 mg alendronate once a week for another 6 months; while in the ALN group, participants were only receiving 70 mg alendronate once a week for the whole study period. Besides, all participants in those two groups were supplementarily prescribed 1200 mg calcium carbonate and 800 IU vitamin D for the whole 12 months. All participants were checked for BMD and bone metabolism (Serum N-propeptide of type I collagen (PINP),β-C-terminal telopeptide of type I collagen (CTX)) before and every 3 months after surgery.

This study had been registered on the website of chictr.org.cn (chictr18000016971) and approved by our institute (SZNSYY2018019). The performance of this study was in concordance with the Declaration of Helsinki, and informed consent regarding the whole process of this study and data publishing permission was acquired from all the participants included.

### Data collection

Data collection was conducted in the orthopedic ward with each patient and patient’s relatives using semi-structured questionnaires for baseline data. Data collection was done after providing informed consent. The questionnaire included sociodemographic characteristics such as name, sex, and age, associated comorbidities and some nutrition habits. In addition, clinical bone metabolism and bone mineral density (BMD) were checked every 3 months on a specified form. If the refracture of patient occurred during follow-up, X-ray and magnetic resonance imaging (MRI) should be used to confirm a fresh fracture.

### Measurement

Bone mineral density of the posterior–anterior lumbar spine, total hip and femoral neck was measured by dual X-ray absorptiometry with a Hologic Discovery A (Hologic, Waltham, MA, USA) in two hospitals. All scans of an individual participant were done on the same densitometer parameter. Quality control measurements were done daily with a Hologic anthropomorphic spine phantom. Our standard deviations of in-vivo same-day reproducibility are 0.005 g/cm^2^, 0.006 g/cm^2^, and 0.007 g/cm^2^ for posterior-anterior spine, total hip, and femoral neck bone mineral density measurements, respectively.

Fasting morning blood samples were obtained at every visit. Serum N-propeptide of type I collagen(PINP) and β-C-terminal telopeptide of type I collagen (CTX), a marker of bone resorption, was measured via a fully automated electro-chemiluminescent immunoassay (Roche Cobas e801 Analyzer) with an interassay coefficient of variation (CV)of 2.9% and 3.2%, respectively, in two hospitals.

### Assessments and safety

The demographic characteristics at baseline including age, preoperative BMD, biochemical markers of bone turnover, and body mass index (BMI) were accordingly recorded in each group. Bone mineral densities at spine, femoral neck, and total hip, respectively, at preoperation, 3 months, 6 months, 9 months, and 12 months were confirmed by dual-energy X-ray absorptiometry. Serum N-propeptide of type I collagen(PINP) and β-C-terminal telopeptide of type I collagen (CTX), which represented the biochemical markers of bone turnover, were also measured by fasting blood at each time visit to the clinic, respectively.

Adherence to treatment was assessed by written diary, unused tables and cartridges were also retrieved to check the patients’ adherence to the treatment and to clarify whether any adverse events occurred. Furthermore, if refracture was suspected, X-ray and magnetic resonance imaging (MRI) were leveraged to figure out this suspicion.

### Statistical analyses

SPSS software 21.0 (IBM, Chicago, USA) was used to analyze the statistics of the parameters in this study. Collected parameters were first analyzed by Kolmogorov–Smirnov’s test to detect if the parameters were normally distributed. The values of BMD, PINP, and CTX at each time point between TPTD + ALN group and ALN group were analyzed by independent student’s *t* test if those parameters were normally distributed, otherwise Mann–Whitney *U* test was used. Moreover, the values of BMD, PINP, and CTX within group at preoperation were also compared to the values at 12 months to observe if any significant changes could be detected when the treatment interval was finished, and those within group changes were analyzed by paired *t* test or Wilcoxon test according to their Kolmogorov–Smirnov’s tests, respectively. The refracture rates between those two groups were compared with Fisher’s exact test. Based on the calculation with PASS software 21.0, a SD of 0.05 and a sample size of 31 patients in each group would reach a power of 80% to detect at least a change of 0.04 g/cm^2^ in BMD. The data in this study were presented as mean ± SD, and a *p* value < 0.05 was considered to be statistically significant.

## Results

### Baseline characteristics

**Fig. 1:**
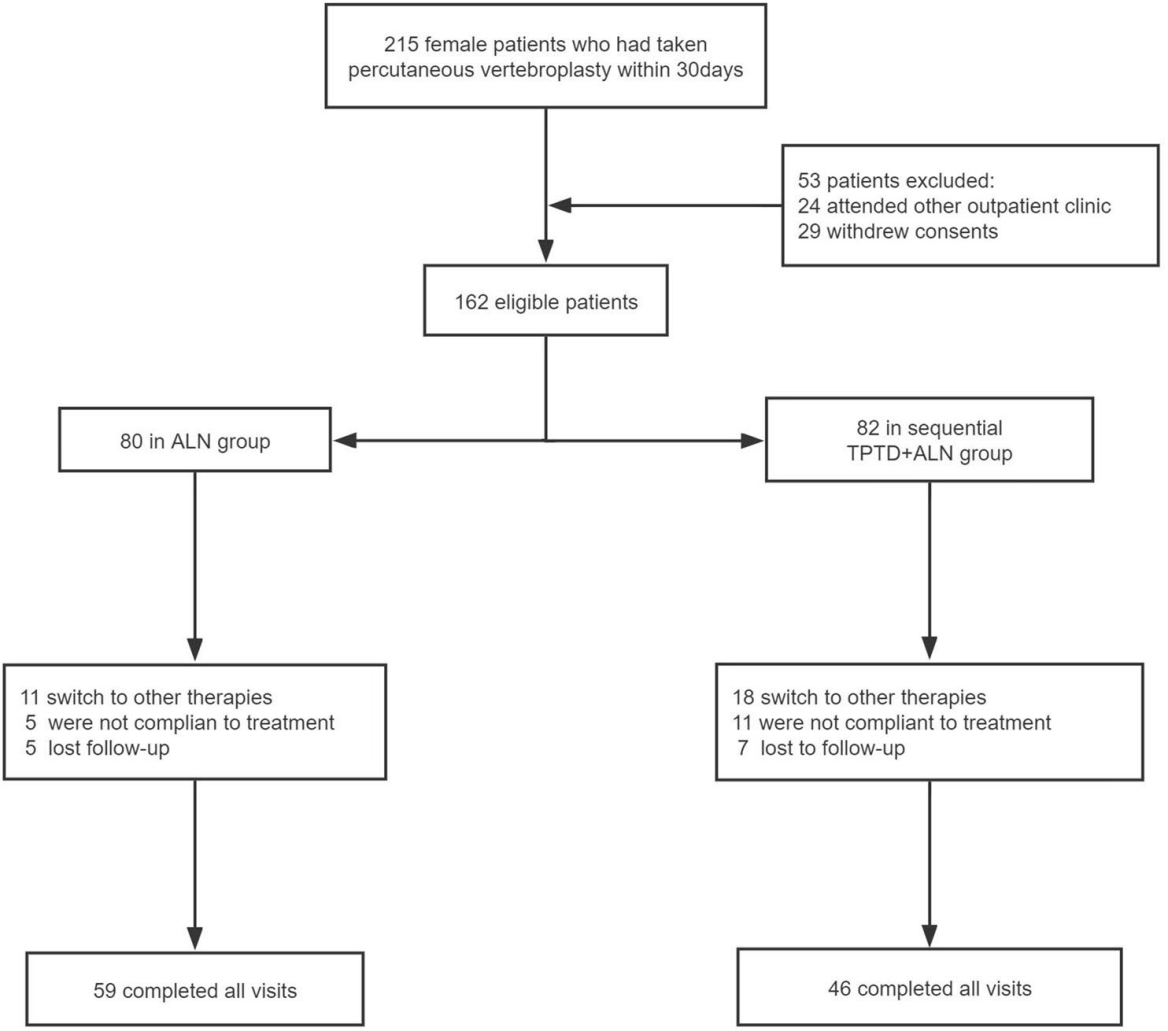
The flowchart of this study

Initially, 215 female patients were primarily screened to meet the inclusion criteria in this study. We finally recruited 162 subjects for the study. In addition, 53 participants were excluded due to attending to other outpatient clinic (*n* = 24) or not agreed to participate (*n* = 29). Patients were assigned to TPTD + ALN group (*n* = 80) and ALN group (*n* = 82).During the treatment period, 57 patients withdrew form this study due to switching to other therapies affecting bone metabolism (*n* = 29), non-compliance (*n* = 16), or lost to follow-up (*n* = 12). Finally, a total of 105 women completed this study, with 46 in TPTD + ALN group and 59 in ALN group, and the flowchart of this study is shown in Fig. 1.

A total of 105 patients were included in the study. The baseline characteristics of those parameters at preoperation were also analyzed and compared, no significant differences were detected, the details of those parameters are shown in Table 1.

**Table 1:** Baseline characteristics

	TPTD + ALN group (*n* = 46)	ALN group (*n* = 59)	*p* value
Age (year)	67.5 ± 4.934	67.39 ± 4.605	0.906
BMD (g/cm^2^)			
Lumbar	0.569 ± 0.069	0.567 ± 0.064	0.932
Femoral	0.382 ± 0.05	0.394 ± 0.053	0.245
Total hip	0.559 ± 0.081	0.584 ± 0.063	0.076
Serum calcium (mmol/l)	2.312 ± 0.167	2.266 ± 0.157	0.073
25-Hydroxyvitamin D(ng/ml)	22.813 ± 5.996	22.671 ± 5.671	0.874
PTH (pmol/l)	3.482 ± 1.151	3.554 ± 1.248	0.675
Bone turnover markers (ng/ml)			
P1NP	41.756 ± 5.391	42.942 ± 7.316	0.359
CTX	0.468 ± 0.1	0.453 ± 0.118	0.499
BMI (kg/m^2^)	20.207 ± 1.715	20.188 ± 1.697	0.956
Vertebral fractures			
Lumbar	29	35	
Thoracic	17	24	0.698
Vertebral fracture type			
Genant I	14	19	
Genant II	32	40	0.846

*BMD* bone mineral density, *PTH* parathyroid hormone, *P1NP* serum procollagen type I N-terminal propeptide, *CTX* serum C-terminal cross-linking telopeptide of type I collagen, *BMI* body mass index, *TPTD + ALN group* sequential treatment of teriparatide followed by alendronate group, *ALN* *group* alendronate alone group

### Refracture rate after percutaneous vertebroplasty in osteoporosis

Suspicious newly formed fractures were confirmed by X-ray and MRI. Totally, 17 refractures were recorded, with 3 in TPTD + ALN group and 14 in ALN group in Table 2. We classified those fractures as vertebral fracture or non-vertebral fracture (other fracture) as vertebrate were our dominatingly cared interest. In TPTD + ALN group, 1 refracture occurred in vertebral while 2 occurred in other part; in ALN group, 8 refractures were confirmed in vertebrae, while 6 in other parts. Percutaneous vertebroplasty was performed in 9 patients with recurrent vertebral fractures both in TPTD + ALN group and ALN group. Other fractures, included 2 distal radius fractures, 2 femoral neck fractures,3 intertrochanteric fractures, and 1 lateral malleolus fractures. Subsequently 2 femoral neck fractures were treated with total hip replacement, 3 intertrochanteric fractures were treated with intramedullary nail fixation, 2 distal radius fractures and 1 lateral malleolus fracture were treated with plaster fixation.

**Table 2:** The comparison of refracture numbers

	TPTD + ALN group (*n* = 46)	ALN group (*n* = 59)	*p* value
Total newly fractures	3 (6.5%)	14 (23.7%)	0.015*
Vertebral fracture	1 (2.2%)	8 (13.6%)	0.038*
Other fracture	2 (4.3%)	6 (10.2%)	0.231

*Denotes statistical significance

### Changes in BMD between TPTD + ALN group and ALN group in follow 12-month follow-up

**Fig. 2:**
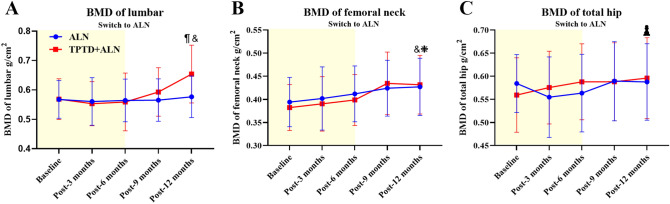
The mean changes of BMD at lumbar (**A**), femoral neck (**B**), and total hip (**C**). ^¶^*p* < 0.001, vs. BMD in ALN; ^&^*p* < 0.001, vs. BMD at baseline in TPTD; ^❈^*p* < 0.01, vs. BMD at baseline in ALN; ^¤^*p* < 0.05, vs. BMD at baseline in TPTD

In the first 6 months, the BMD at lumbar site was not elevated or even decreased slightly compared to the BMD at baseline in both groups. However, when the regime switched to alendronate for the rest 6 months, the BMD in TPTD + ALN group gradually increased. At 12 months, the BMD in TPTD + ALN group was significantly higher than that in ALN group (*p* < 0.001). Furthermore, we also compared the BMD at 12 months with the BMD at baseline within TPTD + ALN group, and found that the BMD at 12 months was also much higher compared to the value at baseline (*p* < 0.001), see Fig. 2A.

The curve of BMD changes at femoral neck was obviously different from that in lumbar. BMD values in both groups were gradually increased after the interventions initiated, however, no statistical differences were detected in either time point during this 1-year follow-up (*p* > 0.05). But when the BMDs at 12 months were compared with BMDs at baseline, both groups revealed significant changes (*p* < 0.001 in TPTD + ALN group, *p* < 0.01 in ALN group), see Fig. 2B.

Even though both the BMDs at lumbar and femoral neck were significantly changed between groups or between baseline and 12 months. The changes of BMDs in total hip were different story. As the changes of BMDs in femoral neck, no statistical differences could be confirmed between TPTD + ALN group and ALN group. Within group comparison, only slight changes could be detected between baseline and 12-month time point (*p* < 0.05), see Fig. 2C.

### VAS scores between TPTD + ALN group and ALN group in 12-month follow-up after percutaneous vertebroplasty

**Fig. 3:**
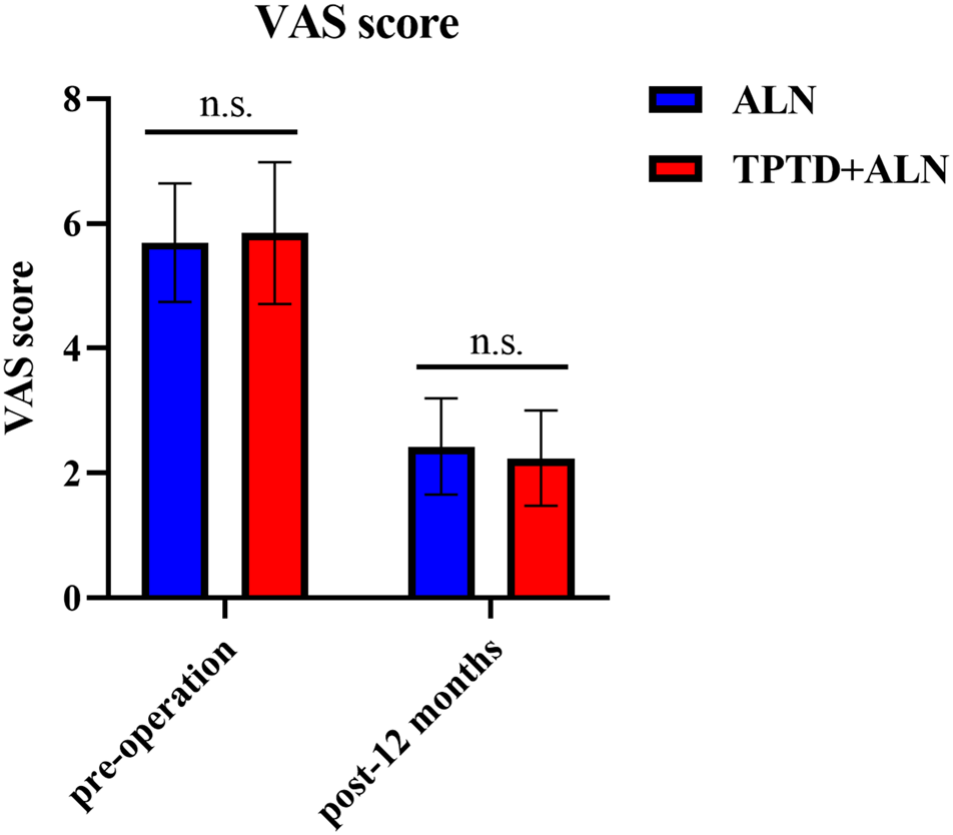
The VAS score at baseline and 12 months, *n.s*. denotes not significant

The VAS scores in those two groups were also analyzed accordingly. There were no significant changes were detected between groups both baseline and 12 months (*p* > 0.05). Compared with baseline, VAS scores at 12 months were significantly lower in both TPTD + ALN and ALN groups (*p* < 0.05), see Fig. 3.

### Changes in biochemical markers of bone turnover between TPTD + ALN group and ALN group in 12-month follow-up

**Fig. 4:**
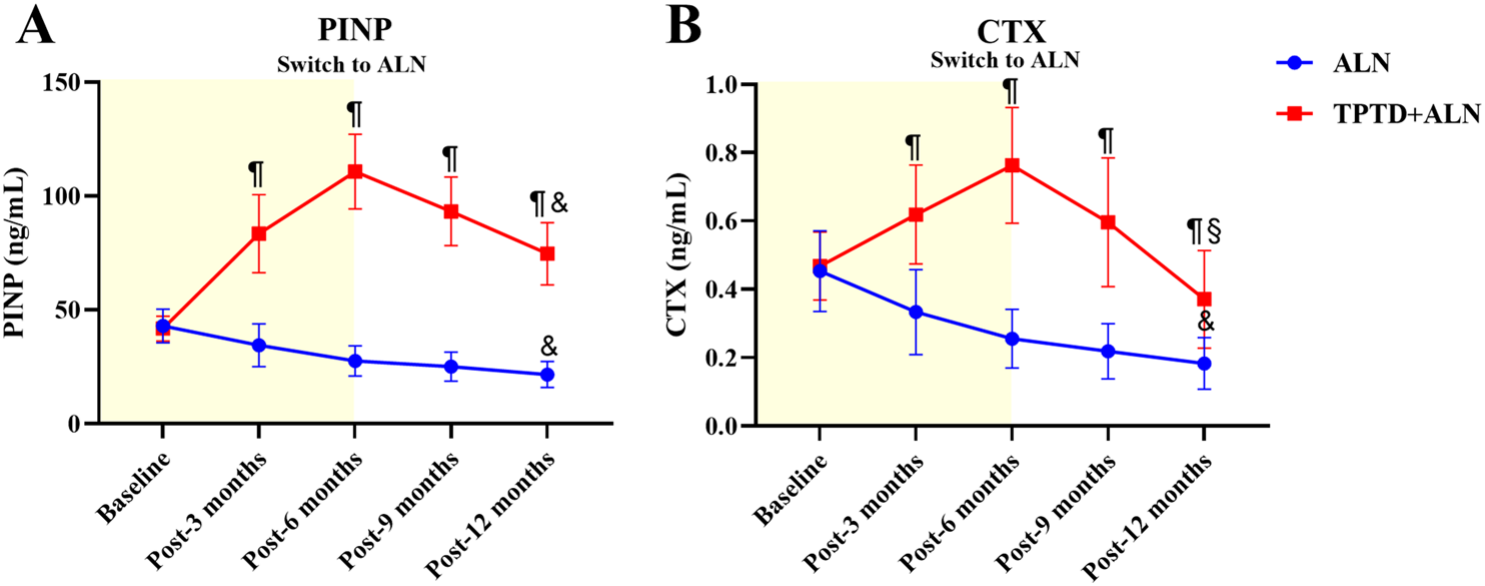
The biochemical markers of bone turnover. **A** The curve of PINP changes; **B** the curve of CTX changes; ^¶^*p* < 0.001, vs. ALN; ^§^*p* < 0.01, vs. PINP value at baseline in TPTD; ^&^*p* < 0.001, vs. CTX value at baseline in ALN

The expression changes of PINP and CTX were observed, respectively. After the administration of teriparatide, PINP and CTX all elevated compellingly. However, the teriparatide was discontinued after 6-month therapy and switched to alendronate, the values of both PINP and CTX were decreased. After statistical analyses, both the expressions of PINP and CTX at 3 months, 6 months, 9 months, and 12 months in TPTD + ALN group were substantially higher than those in ALN group (*p* < 0.001). Moreover, when comparing the expressions of PINP and CTX between baseline and 12 months correspondingly within TPTD + ALN group and ALN group, noticeable changes were also observed, in which both the expressions of PINP and CTX at 12 months in ALN group were significantly reduced when compared to the expressions at baseline (*p* < 0.001). Differently, the expression of PINP at 12 months was still higher than that at baseline in TPTD + ALN group (41.76 ± 5.40 vs. 74.64 ± 13.68, *p* < 0.001), while the CTX at 12 months decreased significantly, even below the initial value at baseline (0.47 ± 0.10 vs. 0.37 ± 0.14, *p* < 0.01), seen Fig. 4.

## Discussion

Osteoporotic vertebral compression fracture (OVCF) is one of the many complications due to osteoporosis. Mild external or internal stresses, such as the stress caused by bend or cough, might predispose these patients to develop OVCF. Although percutaneous vertebroplasty (PVP) and percutaneous kyphoplasty (PKP) can effectively restore the vertebrate height and relieve pain rapidly, refractures after surgery are commonly seen. As reported, 14.6% OVCF patients would sustain refracture after PVP procedure [18]. Plenty factors can be accountable, including old age, inherent comorbidity, improper operation, low bone mineral density, etc. [19–21]. Among them, low bone mineral density may be the most dominating factor [22]. Therefore, improving bone density through anti-osteoporosis therapy can be an independent consideration for prevention of refracture in these patients [23].

Teriparatide, the first approved anabolic anti-osteoporotic agent, has shown to improve the bone quality and bone mass in postmenopausal women, specifically by elevating BMD and trabecular thickness [13]. Comparatively, studies had shown that patients who received teriparatide had achieved a lower vertebral fracture rate than counterparts who received risedronate [24, 25]. However, the efficacy of teriparatide for osteoporosis would be decreased after the discontinuation of this agent, in which the BMD would gradually be down-regulated after the cessation of teriparatide. Besides, the daily administration and big cost would also predispose the patients to discontinue [13, 26]. Therefore, some modifications should be adopted.

In this study, we sequentially administered the teriparatide for the first 6 months which were then followed by the alendronate for another 6 months. We hoped this regime modification could not only reduce the cost by long-period prescription of teriparatide, but also could consolidate or even elevate the BMD after cessation of this regime. Fortunately, refracture rate in TPTD + ALN group was 6.5%, greatly lower than ALN group. The incidence of new vertebral fractures was 2.2% in the TPTD + ALN group compared with 13.6% in the ALN group. These results indicate that the short-sequentially TPTD-ALN therapy has a good effect on the protection of refractures after percutaneous vertebroplasty. This is similar to the effect of long-term sequentially parathyroid hormone and alendronate therapy [27].

One of main factors associated with refracture after percutaneous vertebroplasty was BMD. In this study, at 12-month time point, the BMD at lumbar in TPTD + ALN group was much higher than that in ALN group, while this difference was not observed in the BMD at femoral neck and total hips. Those results showed that teriparatide followed with alendronate was in particular effective in elevating the BMD in spine. This observation was consistent with the study by Yasuaki and his colleagues [14]. However, the BMD in the femoral neck was also significantly increased in their study at 12 months, slightly different with our findings. The reason might be that the patients in their group had taken prior bisphosphonate treatment.

Nevertheless, we also observed that the PINP and CTX significantly increased in the first 6 months when teriparatide was administered, which meant bone metabolism was mobilized, both in anabolism and catabolism. This is similar to studies that PINP and CTX got increasing during 6–12 months of teriparatide [28]. In the first 6 months of PTH treatment, PINP expression increased rapidly and reached a peak at about 6 months, and then the PINP expression curve tended to be flat [28]. This indicated that PTH bone formation might peak at 6 months. It was described by Cusano and his colleagues that concept of the anabolic time window when PTH directly stimulates bone formation before stimulation of bone remodeling [29].

When alendronate was administered, the anabolic and catabolic effect were both down-regulated, which were reflected by the decrease in the values of PINP and CTX from 6 to 12 months. However, at 12 months, the expression of PINP was still higher compared to that at baseline while the expression of CTX had fell below baseline. Such results meant the anabolic activity of bone was still higher than the catabolic activity during this process, and this might be the explanation why the BMD at lumbar was increased and why the refracture rate was decreased during this period. The short-sequential treatment of teriparatide and alendronate, not only retained the osteogenic effect of PTH, but also made use of the osteoclastic effect of ALN, achieved the dual effects of osteogenic and osteoclastic inhibition at 12 months. However, why the elevation of BMD at lumbar was much higher than total hip and femoral neck was still elusive and needed to be investigated in future studies.

This study also had some limitations. Firstly, the sample size was small and follow-up periods were not long enough, which might bring this study some biases, including missing some refracture patients; second, the participants in this study were generally assigned by their only choice, which meant randomization was not matched strictly by their hierarchy of ages, T scores, sexes, or occupations. However, this study also had some strengths. This study valuably revealed that prescription of alendronate would be beneficial for prevention of BMD loss after the cessation of teriparatide, and this effect was due to the elevated anabolic metabolism by teriparatide and reduced catabolic metabolism by alendronate, which altogether lead to the relatively low refracture rate in TPTD + ALN group.

## Conclusions

In this 1-year study, short-sequential administration of teriparatide followed by alendronate was more effective in elevating the BMD compared to the counterpart with alendronate alone, and the gains in BMD finally resulted in reduced refracture rate in osteoporotic patients after percutaneous vertebroplasty. The short-sequential treatment of teriparatide and alendronate maybe a cost-effective strategy for prevention of refracture after percutaneous vertebroplasty in osteoporosis.

## Data Availability

The data that support the findings of this study are available from the corresponding author upon reasonable request.
